# Joint effect of particulate matter and cigarette smoke on women’s sex hormones

**DOI:** 10.1186/s12905-021-01586-w

**Published:** 2022-01-08

**Authors:** Anna Merklinger-Gruchala, Grazyna Jasienska, Inger Thune, Maria Kapiszewska

**Affiliations:** 1 Faculty of Medicine and Health Sciences, Andrzej Frycz Modrzewski Krakow University, ul. Gustawa Herlinga-Grudzińskiego 1, 30-705 Kraków, Poland; 2 Department of Environmental Health, Faculty of Health Sciences, Jagiellonian University Medical College, ul. Skawińska 8, 31-066 Kraków, Poland; 3 Department of Oncology, Oslo University Hospital, Ullevål, 0424 Oslo, Norway; 4 Institute of Clinical Medicine, University of Oslo, Oslo, Norway; 5 Institute of Clinical Medicine, UIT The Arctic University of Norway, 9019 Tromsö, Norway

**Keywords:** Air pollution, Particulate matter, PM10, Cigarette smoke, Polycyclic aromatic hydrocarbons, PAH, Estradiol, Estrogens, Progesterone, Gonadal Steroid Hormones, Menstrual cycle, Luteal phase, Reproductive health

## Abstract

**Background:**

Although relationships between exposure to air pollution and reproductive health are broadly studied, mechanisms behind these phenomena are still unknown. The aim of the study was to assess whether exposure to particulate matter (PM10) and tobacco smoking have an impact on menstrual profiles of 17β-estradiol (E2) and progesterone (P) and the E2/P ratio.

**Methods:**

Levels of sex hormones were measured daily in saliva during the entire menstrual cycle among 132 healthy, urban women. Exposure to smoking (active or passive) was assessed by questionnaire, whilst exposure to PM10 with municipal monitoring data.

**Results:**

During the early luteal phase, profiles of E2 were elevated among women with higher versus lower exposure to PM10 (*p* = 0.02, post-hoc tests). Among those who were exposed versus unexposed to tobacco smoking, the levels of mean E2 measured during the entire cycle were higher (*p* = 0.02). The difference in mean E2 levels between the group of joint exposure (i.e. to high PM10 and passive or active smoking) versus the reference group (low PM10, no smoking) was statistically significant at *p* = 0.03 (18.4 vs. 12.4 pmol/l, respectively). The E2/P ratios were higher among women with higher versus lower exposure to PM10 and this difference was seen only in the early luteal phase (*p* = 0.01, exploratory post-hoc tests).

**Conclusions:**

We found that PM10 and tobacco smoking affect ovarian hormones independently and do not interact with each other. Both exposures appear to have estrogenic effects even though women's susceptibility to these effects differs across the menstrual cycle. We propose that the hormonal mechanisms are involved in observed relationships between air pollution and smoking with women’s reproductive health.

## Background

Ambient particle matter and tobacco smoke, in addition to being a contributor to air pollution, are considered to be the world’s largest environmental health threats due to its role in increased incidence, morbidity and mortality of many diseases [1, 2]. Particularly, exposure to both pollutants may lead to adverse reproductive and perinatal problems, such as infertility, subfecundity, variable menstrual cycle length, miscarriage, and stillbirth, as well as other poor pregnancy outcomes, suggesting strong impact on reproductive function in women [3–7].

Many aspects of a women’s reproductive health depend on the level and ratio of the two main female sex hormones—17β–estradiol (E2) and progesterone (P) [8]. In addition, E2/P imbalance, known as the “unopposed estrogen hypothesis”, negatively affects embryo implantation and has been used as a predictor in the outcome of assisted reproductive techniques [9, 10]. Further, any disturbances in the concentration of these circulatory sex hormones can lead to a disruption in the reproductive functions mentioned above and enhance the risk of hormone-dependent cancers [11–16].

Some elements in the complex mixture of chemicals, consisting of the volatile or semi-volatile compounds in the gas phase or attached to the respirable particulate matter (PM) and cigarette smoke, are identified as environmental endocrine disrupting chemicals (EDC) [17–19]. The correlation between concentrations of PM10 and PM10-bound polycyclic aromatic hydrocarbons (PAHs) was found to be correlated [18–22]. Some PAHs have been reported to act as agonists while others as antagonists of estrogens [23, 24] and, thus, influencing ovarian estrogens levels. Prolonged exposure to air pollution and cigarette smoke containing PAHs compounds may affect the amount of estrogens in a woman’s body over the course of her life, resulting in an increased risk of breast cancer [11–16]. Breast cancer risks have been shown to be elevated in urban areas where air pollution levels are higher [25]. Furthermore, an excessive amount of E2 without concomitantly high levels of P seems to be responsible for an increased risk of endometrial cancer [26, 27].

Given the similarities in the biological mechanisms of both types of pollutants, it is plausible that the widespread exposure to a combination of ambient particle matter (PM10) and cigarette smoke may impact the level of E2 in women residing in an environment with higher levels of pollutants. In line with these findings, we sought to assess whether the joint exposure to PM10 and active/secondhand smoking acts additively on sex hormone concentrations. We also investigated the separate effects of PM10 and tobacco smoke, a surrogate for exposure to PAHs, on daily measurements of salivary levels of unbound, bioactive E2 and P collected during one menstrual cycle in regularly cycling, healthy premenopausal women.

## Methods

### Study participants

One hundred and thirty six Polish urban women between 24 and 35 years of age were recruited by advertisements between June 2001 and June 2003. They were able to participate in the study if they had regular menstrual cycles, no fertility problems, and gynecological and chronic disorders (i.e. diabetes, hypo/hyperthyroidism), did not take any hormonal medication or use hormonal contraception and had not been pregnant or lactating during the 6 months before recruitment.

### Hormonal measurements

During one entire menstrual cycle each woman collected daily morning saliva samples to assess the levels of 17-β estradiol (E2) and progesterone (P). Morning saliva samples were collected daily throughout the entire menstrual cycle by participants beginning on the first day of menstruation. After waking up, each women collected saliva samples in plastic tubes pretreated with sodium azide following published protocols [28]. For the ease of collecting saliva, participants were provided with laboratory tested chewing gum. Samples were stored in a refrigerator until the date of shipment to the Laboratory of Reproductive Ecology, Harvard University, where the samples were analyzed. At the laboratory, all samples were stored at -280C and thawed at the time of analysis. Incomplete collection or loss during laboratory procedures were responsible for only 5.3% of daily samples missing [29].

Both hormones were analyzed using radioimmunoassay method with published modifications to the manufacturer’s protocol [30]. Estradiol measurements were performed with I-125-based radioimmunoassay kit (#39100, Diagnostic Systems Laboratories, Webster, TX, USA) I-125 (#39100, Diagnostic Systems Laboratories, Webster, TX, USA). Progesterone measurements were performed with I-125 based radiomunoassay kit (#3400, Diagnostic Systems Laboratories, Webster, TX, USA).

Saliva samples from 20 days (reverse cycle days—5 to—24) of each cycle were analyzed for the concentration of E2. In order to estimate the day of ovulation, cycles were aligned on the basis of identification of the day of the midcycle estradiol drop (day 0), according to the published methods [30]. The mean E2 values from 18 consecutive days of each menstrual cycle aligned on day 0 (i.e. between days − 9 and + 8) were used in the analyses because of higher variation in E2 measurements found at the beginning and at the end of the cycles.

Concentrations of P were analyzed in samples aligned on day 0 (pointed out in the profiles of E2), starting from the day 2 and up to day 8 of the luteal phase. Thus 7 consecutive daily samples of the luteal phase of each cycle were assayed for P.

### Particulate matter exposure assessment

To assess the exposure to ambient air pollution during the menstrual cycle for each woman, municipal ecological monitoring data was used. The raw ambient air quality data was primarily extracted from the State Environmental Monitoring the system maintained by the Inspector for Environmental Protection of the Malopolska Region. Exposure measurements of particles with an aerodynamic diameter of ≤ 10 μm PM10 [μg/m^3^] were taken from all available monitoring stations (five in year 2000, four in year 2002 and three in year 2003), covering most of the areas of the Krakow city. The raw measurements of PM10 were available as 1 h averages and were available for 84%, 84% and 96% of all working monitoring stations in years 2000, 2002 and 2003, respectively. The hourly measurements of PM10 were averaged arithmetically across all monitoring stations and calculated as 24-h averages (between 6 a.m. the given day of taking of saliva sample to 7 a.m. the day before).

### Smoking exposure assessment

The current smoking status (yes or no) and current exposure to passive smoking at home (yes or no) was collected with a general questionnaire (partly administered by an interviewer and partly self-reported).

### Other measurements

A general questionnaire was also used to collect information on education, sociodemographic factors (such as age, marital status), reproductive history (age at menarche, age at first birth and parity) as well as past use of hormonal medication. The current menstrual cycle length during saliva sample collection was reported with a structured diary.

Anthropometric measurements (body height, body weight, and body fat %) were taken twice for every participant. A detailed description of anthropometric methods was published previously [30].

### Statistical analysis

Participants were categorized into two groups of PM10 based on their exposure during the entire menstrual cycle (lower and higher exposure) according to PM10 median value.

Due to the association between smoking status and passive smoking, the dichotomous variable “Total Smoke Exposure” (“TSE”), describing total exposure to tobacco smoke was calculated. Women were categorized as being exposed either as an actual smoker or a passive smoker (category: “smoke”, “SM”) or as not being exposed neither as an active nor passive smoker (category: “non-exposed to smoke”, “nSM”).

In order to investigate the combined effect of exposure to PM10 and smoking, women were divided into 4 groups: low exposure to PM10 and non-exposed to smoke (“LowPM-nSM”, the reference condition), high exposure to PM10 and non-exposed to smoke (“HighPM-nSM”), low exposure to PM10 and exposed to smoke (“LowPM-SM”), and high exposure to PM10 and exposed to smoke (“HighPM-SM”).

Descriptive statistics and frequency distributions were used to summarize the characteristics of participants. Differences among PM10 and Total Smoke Exposure groups in potentially confounding factors, including anthropometric, reproductive, and lifestyle characteristics were tested with simple t-tests with PM10 groups as grouping variable. For not equal variances, the t-test for separate variance estimates were applied. To compare differences in frequencies of nominal potential confounders between PM10 and Total Smoke Exposure groups, a chi-square test was performed.

To assess the association between two exposures: PM10 exposure and smoking, and hormonal profiles, a repeated measures analysis of covariance (RM-ANCOVA) with group-by-time interaction terms was used. Adjusted univariate significance tests were carried out, because our data did not meet the assumption of sphericity (Mauchly criterion close to zero, *p* < 0.001). To account for the within-subject correlation, the Huynh and Feldt adjustment (H–F adjustment) for F test was applied.

In order not to exclude women with missing values of hormonal data from the RM-ANCOVA, linear interpolation was used. For participants that skipped no more than 2 consecutive days of the menstrual cycle, missing value (or values) was calculated as a mean of two neighboring measurements, whilst for the missing values appeared at the end of the interval, the value from the day next to the missing value was used. The number of estimated E2 values in the entire group of women was 6%, whilst *p* values—below 5%.

The E2/P ratio during each day of the luteal phase (between day 2 and day 8) was calculated. The hormonal measurements design was shown on Table 1.

**Table 1:** The frequency of hormonal measurements of the study

Phases	Mid-follicular					Late follicular			Ovulatory			Early luteal			Mid-luteal			
Days of the cycle	− 9	− 8	− 7	− 6	− 5	− 4	− 3	− 2	− 1	0	1	2	3	4	5	6	7	8
Estradiol (E2) measurements	X	X	X	X	X	X	X	X	X	X	X	X	X	X	X	X	X	X
Progesterone (P) measurements												X	X	X	X	X	X	X
E2/P ratio calculations												X	X	X	X	X	X	X

The “X” represents the day of sample collection or E2/P ratio calculation

Two dichotomous variables: groups of PM10 exposure (low and high) and Total Smoke Exposure (nSM and SM) were entered as between-subject factors, whilst hormone levels (previously logarithmically transformed) as within—subject factor of the repeated measures, called “time” (with 18 levels for E2, with 7 levels for P and E2/P ratio). Thus, possible differences in hormonal profiles across PM10 and Total Smoke Exposure groups were to be explored. Because we found that the PM10 levels correlated with age, which in turn can be linked with sex steroids levels, age was entered in the final models as confounder. Moreover, since menstrual cycle length is known from the literature to be important confounder of the association between particulate matter and ovarian hormones, this additional factor was also included in the repeated measures ANCOVA models.

All hormonal data were logarithmically transformed before entered to the models. Because the interaction between "time" and PM10 groups appeared to be statistically significant, an exploratory post-hoc analyses were performed. Then, a contrast test between low and high PM10 groups was used to further explore the association between pollution and sex hormones during particular phases of the cycle. In order to do so, the cycle was divided into phases: mid-follicular (days from − 9 to − 5), late follicular (days from − 4 to − 2), ovulatory (days from − 1 to 1), early luteal (days from 2 to 4) and mid-luteal (days from 5 to 8). These contrasts analysis were only exploratory in nature, and thus did not needed standardization to multiple comparison [31].

Next, we investigated whether hormonal response of PM10 (low vs. high) differs across the groups of Total Smoke Exposure (nSM vs. SM) by incorporating an interaction terms (Total Smoke Exposure × PM10 groups) and (Total Smoke Exposure × PM10 groups × Time) into the final RM-ANCOVA models.

The joint effect of both exposures i.e. PM10 and smoke on E2 levels was investigated by building a new model of repeated measures ANCOVA with 4 groups as between-subject factor: “HighPM-nSM”, “LowPM-SM”, “HighPM-SM”, and “LowPM- nSM”, treated as the reference condition, and E2 (with 18 levels) as within-subject factor of the repeated measures, called “time”, with cycle length and age as continuous confounders. This model allows for reporting the separate effect of each exposure as well as the joint effect compared with the unexposed group as a joint reference category, what follows the STROBE (Strengthening the Reporting of Observational Studies in Epidemiology) [31].

Additionally, adjusted linear trend was tested by treating 4 groups of women (“LowPM- nSM”,“HighPM- nSM”, “LowPM- SM”, and “HighPM- SM”) as a single ordinal variable in adjusted model. Results were considered statistically significant at a *p* value < 0.05. Statistica 13.0 software was used for all statistical analyses.

Based on the observed effect sizes, a post-hoc power analysis was performed. Power analysis was conducted retrospectively because there were no prior research data in the literature to project data variance prospectively.

### Study sample

Out of 136 women who collected saliva samples, we excluded 4 women, because of missing data about the date of the cycle and thus impossibility of assessing the pollution exposure. Performing repeated measures analyses were possible for women for whom a reliable identification of the day of the mid-cycle E2 drop could be made, and for whom a full profiles (including profiles of interpolated values) were present. Full E2 profiles aligned on Day 0 (for 18 cycle days) were available for n = 117 women, full P profiles (for 7 cycle days) were available for n = 120 women, whilst full E2/P ratios profiles (for 7 cycle days) were available for n = 120 women. The number of women analyzed in a final models may vary because of missing values in confounding factors.

## Results

### General characteristics of women

Age of participants ranged from 24 to 35 years (Mean = 29.5). Among all women participating in the study, 60.3% reported to be ever married. The mean age of menarche was 13.2 years (SD = 1.4). The usual cycle length was 29 days ≤ among 50% of the participants with a range between 24 and 39 days. At the time of the study, 64% women reported to be nulliparous. The mean body weight ranged from 42.1 to 84.6 kg (Me = 58.2), the average body fat content was 25.2%. The average body height equaled 164.5 cm. The median body mass index (BMI) was 21.4 kg/m^2^.

A total of 18.3% of women (n = 24) classified themselves as a current smoker at the time of the study entry, while 27% of the participants reported to live with a smoker. Taking passive and active smoking together, 33.3% of women were exposed to either or both kinds of smoke (SM group), whilst 63.6% remained unexposed (nSM group). The median value of PM10 exposure during the menstrual cycles days was 54.8 μg/m^3^ with a minimum of 29.3 μg/m^3^ and a maximum of 93.6 μg/m^3^.

Reproductive, anthropometric and lifestyle characteristics of women were similar in the two groups of PM10 levels except for participant's age and Total Smoke Exposure (Table 2). The mean age was significantly lower in the group of low versus high exposure to PM10 (28.9 vs. 30.1 years). Among high PM10 group, women were classified more likely as non-exposed to smoke than exposed to smoke (74% vs. 26%, respectively), whilst in the low PM10 group, the difference between non-exposed and exposed to smoke was lower (55% vs. 45%, respectively, Table 2). Reproductive, anthropometric and lifestyle characteristics of women did not significantly differ across Total Smoke Exposure groups (Table 3).

**Table 2:** Anthropometric, reproductive, and lifestyle characteristics of women across PM10 groups

Characteristics	Level	Statistics	PM10 exposure		Test
			Low	High	
Age [years]		N	60	70	t test; df = 128; *p* = 0.032
		Mean	28.9	30.1	
		SD	2.8	3.3	
Age at first child [years]		N	20	30	t test; df = 48; *p* = 0.570
		Mean	24.5	23.9	
		SD	2.3	3.6	
Parity status	No	N	40	40	Pearson Chi-square: 1.2, df = 1, *p* = 0.266
		%	66.7	57.1	
	Yes	N	20	30	
		%	33.3	42.9	
Menarcheal age [years]		N	60	68	t test, separate variance estimates; df = 106; *p* = 0.060
		Mean	13.5	13.0	
		SD	1.6	1.2	
Cycle length [days]		N	61	70	Mann–Whitney U test, *p* = 0.115
		Median	29.0	27.5	
		IQR	26.0–32.0	26.0–30.0	
Height [cm]		N	61	70	t test; df = 129; *p* = 0.715
		Mean	164.3	164.7	
		SD	6.0	6.1	
Body fat [%]		N	60	69	t test; df = 127; *p* = 0.104
		Mean	26.1	24.3	
		SD	5.4	6.5	
Body mass index* [kg/m^2^]		N	61	70	Mann–Whitney U test, *p* = 0.076
		Median	22.5	20.8	
		IQR	20.3–24.3	19.9–23.3	
Body weight [kg]		N	60	70	t test; df = 128; *p* = 0.282
		Mean	60.8	59.1	
		SD	8.1	9.0	
Marital status	Single	N	24	26	Pearson Chi-square = 0.2; df = 1, *p* = 0.681
		%	40.7	37.1	
	Ever married	n	35	44	
		%	59.3	62.9	
Smoking status	Non-smoker	N	43	60	Pearson Chi-square = 3.4, df = 1, *p* = 0.066
		%	74.1	87.0	
	Smoker	N	15	9	
		%	25.9	13.0	
Living with smoker	No	N	38	54	Pearson Chi-square = 2.6, df = 1, *p* = 0.109
		%	65.5	78.3	
	Yes	N	20	15	
		%	34.5	21.7	
Total Smoke Exposure	nSM	N	32	51	Pearson Chi-square = 4.9, df = 1, *p* = 0.027
		%	55.2	73.9	
	SM	N	26	18	
		%	44.8	26.1	

*IQR* interquartile range (Q1–Q3)

*Body mass index was calculated as a participant’s weight (in kilograms) divided by the square of participant’s height (in metres)

**Table 3:** Anthropometric, reproductive, and lifestyle characteristics of women across Total Smoke Exposure groups

Characteristics	Level	Statistics	Total Smoke Exposure		Test
			nSM	SM	
Age [years]		N	84	44	t test; df = 126; *p* = 0.218
		Mean	29.8	29.0	
		SD	3.2	2.9	
Age at first child [years]		N	31	17	t test; df = 46; *p* = 0.055
		Mean	24.7	22.9	
		SD	3.3	2.5	
Parity status	No	N	52	28	Pearson Chi-square: 0.04, df = 1, *p* = 0.849
		%	61.9	63.6	
	Yes	N	32	16	
		%	38.1	36.4	
Menarcheal age [years]		N	82	43	t test; df = 123; *p* = 0.876
		Mean	13.3	13.3	
		SD	1.3	1.6	
Cycle length [days]		N	84	44	Mann–Whitney U test, *p* = 0.625
		Median	28.0	29.0	
		IQR	26.0–31.5	26.0–30.0	
Height [cm]		N	84	44	t test; df = 126; *p* = 0.748
		Mean	164.6	164.2	
		SD	6.0	6.2	
Body fat [%]		N	83	44	t test; df = 125; *p* = 0.944
		Mean	25.2	25.1	
		SD	6.4	5.4	
Body mass index* [kg/m^2^]		N	88	44	Mann–Whitney U test, *p* = 0.475
		Median	21.1	22.1	
		IQR	20.0–23.8	20.0–24.0	
Body weight [kg]		N	84	44	t test; df = 126; *p* = 0.819
		Mean	59.7	60.0	
		SD	8.5	8.7	
Marital status	Single	N	34	15	Pearson Chi-square = 0.38, df = 1, *p* = 0.541
		%	40.5	34.9	
	Ever married	N	50	28	
		%	59.5	65.1	

*Significance was set at *p* = 0.05

### Estradiol profiles across PM10 groups and Total Smoke Exposure groups

**Fig. 1:**
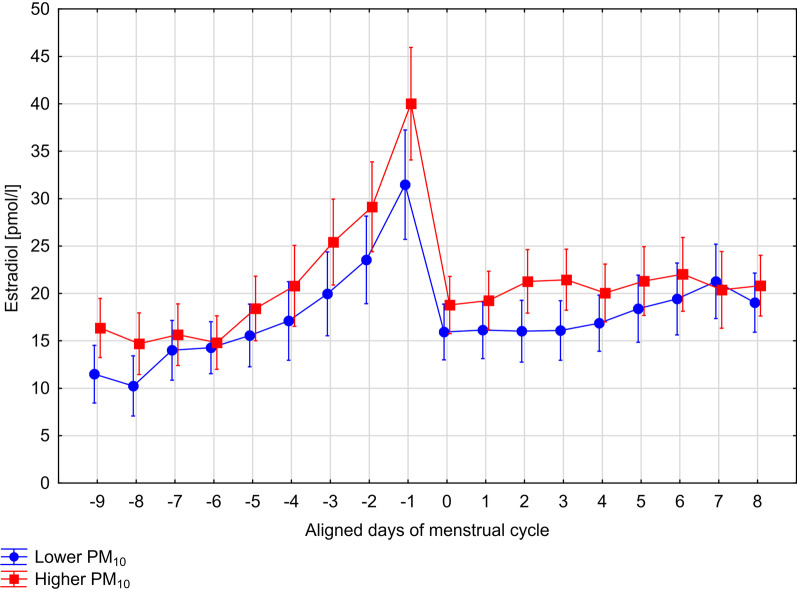
Profiles of 17-b-estradiol in two groups of regularly menstruating women exposed to low (n = 52) and high (n = 61) levels of PM10, after controlling for age, active and passive smoking and cycle length

Among regularly menstruating women, the mean levels of E2 among exposed to higher levels of PM10 were elevated in comparison to those who were exposed to lower PM10 levels, however, the difference was not statistically significant (17.0 pmol/1 vs. 14.3 pmol/l, between-subjects test, F 1,108 = 3.05, *p* = 0.08), after adjustments (Table 4). The same RM-ANCOVA model with Huynh–Feldt corrections showed that the difference in E2 profiles between low and high PM groups varied throughout days of menstrual cycle, as shown by the significant PM10 groups × time interaction term (within-subjects test, F 17,1836 = 1.94, *p* = 0.02, with H–F correction). Profiles of E2 in two groups of women exposed to low and high levels of PM10 are shown in Fig. 1. To further explore the differences in E2 profiles across two exposure groups, we conducted an exploratory post-hoc analyses, separately in each phase of the menstrual cycle. We observed that the differences in E2 profiles between women of high and low exposure to PM10 were seen in the early luteal phase of the cycle. The E2 levels measured during days 2–4 of the luteal phase was elevated when exposure to PM10 was high in comparison to the E2 levels noted when PM10 concentrations was low (F 1,108 = 5.22, *p* = 0.02). There were no statistically significant differences in E2 profiles in other phases of the cycle, i.e. the mid-follicular (F 1,108 = 2.91, *p* = 0.09), the late follicular (F 1,108 = 3.13, *p* = 0.08), the ovulatory (F 1,108 = 3.21, *p* = 0.08), and the mid-luteal (F 1,108 = 0.25, *p* = 0.61).

**Table 4:** Effects of two exposures (PM10 and Tobacco Smoke) on Estradiol (E2), Progesterone (P) levels and E2/P ratio, after standardization to age and cycle length

Variable	Level	Mean	SE	Between-subject effects	Within-subject effects
				*p* value	*p* value (with H–F corrections)
		Estradiol [pmol/l]—entire cycle			
PM10	Low	14.3	1.07	[Ref.]	[Ref.]
	High	17.0	1.07	0.08	0.02
Total Smoke Exposure	nSM	13.8	1.06	[Ref.]	[Ref.]
	SM	17.7	1.09	0.02	0.18
PM10 × Total Smoke Exposure	Interaction effect			0.64	0.41
PM10 × Total Smoke Exposure (4 groups)*	LowPM-nSM	12.4	1.10	[Ref.]	[Ref.]
	HighPM-nSM	15.3	1.07	0.09	0.02
	LowPM-SM	16.5	1.11	0.04	0.17
	HighPM-SM	18.4	1.16	0.03	0.27

		Progesterone [pmol/l]—luteal phase			
PM10	Low	130.4	1.08	[Ref.]	[Ref.]
	High	130.7	1.08	0.99	0.19
Total Smoke Exposure	nSM	129.0	1.07	[Ref.]	[Ref.]
	SM	132.2	1.10	0.84	0.30
PM10 × Total Smoke Exposure	Interaction effect			0.85	0.16

		Estradiol/Progesterone ratio—luteal phase			
PM10	Low	0.1	1.11	[Ref.]	[Ref.]
	High	0.2	1.12	0.11	< 0.01
Total Smoke Exposure	nSM	0.1	1.09	[Ref.]	[Ref.]
	SM	0.1	1.14	0.26	0.23
PM10 × Total Smoke Exposure	Interaction effect			0.80	0.23

Means were derived from calculations on log-transformed values and then back-transformed by taking the antilog

*Model with dummy variables

**Fig. 2:**
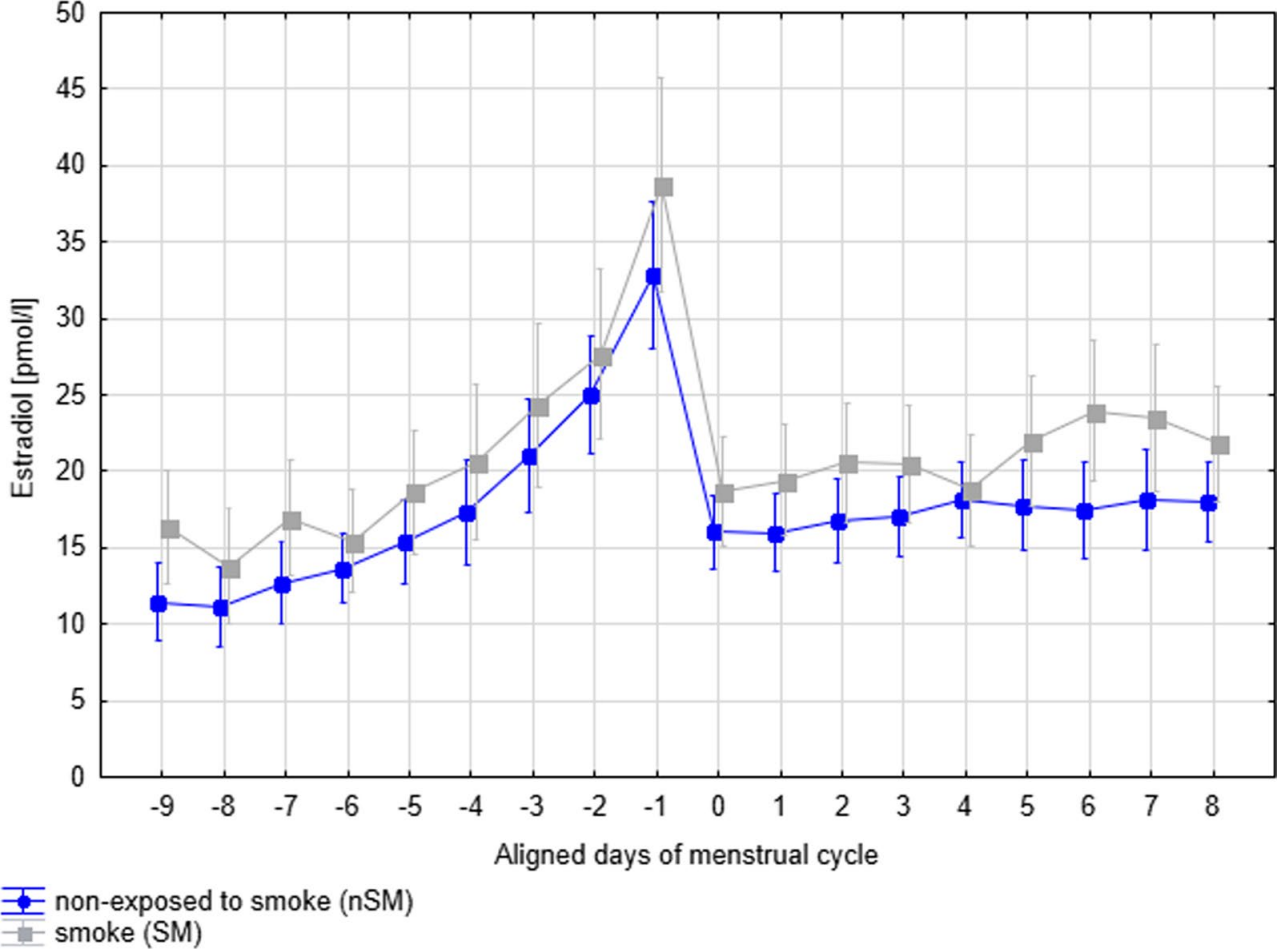
Levels of 17-b-estradiol measured throughout entire menstrual cycle in two groups of women exposed (SM) versus unexposed (nSM) to smoke

Among women belonging to SM group, i.e. exposed to either passive or active smoking, the mean levels of E2 was higher in comparison to the group who were non-exposed to smoking at all—nSM (17.7 pmol/1 vs. 13.8 pmol/l, between-subjects test, F 1,108 = 5.37, *p* = 0.02), after controlling for age, particulate matter and cycle length (Table 4). Levels of E2 measured throughout the entire menstrual cycles in two groups of women exposed (SM) versus unexposed (nSM) to smoking are shown in Fig. 2, where the profile of E2 for SM is elevated in relation to the profile of E2 for nSM for most of the days of the cycle. The same RM-ANCOVA model with Huynh–Feldt corrections indicated that the difference in E2 profiles between exposed to smoke (SM) versus unexposed group (nSM) did not vary throughout days of menstrual cycle, as shown by the nonsignificant Total Smoke Exposure groups × time interaction term (within-subject test, F 17,1836 = 1.35, *p* = 0.18, with H–F correction).

We also found that the effect of PM10 on mean E2 (between-subject test, F 1,107 = 0.22, *p* = 0.64) or E2 profiles (within-subject test, F 17,1819 = 1.03, *p* = 0.41) did not differ in relation to Total Smoke Exposure.

**Fig. 3:**
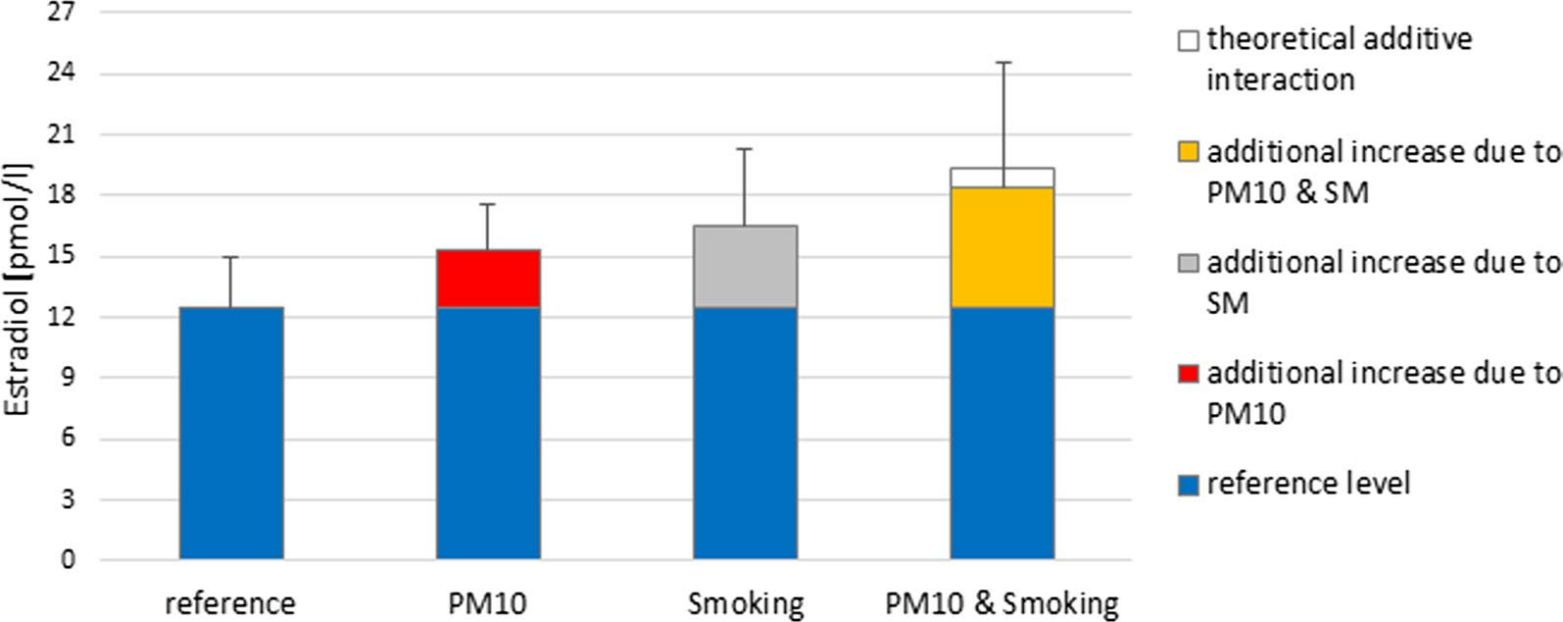
The separate and combined response of both exposures (PM10 and Total Smoke Exposure) on mean E2 levels of entire menstrual cycle

The analysis of joint effects of both exposures i.e. PM10 and smoking on E2 levels indicated that among women exposed to low PM10 and not exposed to smoking (as active or passive smoker at baseline, LowPM-nSM), the average levels of E2 measured throughout menstrual cycle was 12.4 pmol/l. This can be considered as the background level. In women exposed to high PM10 at baseline this was 15.3 pmol/l, resulting in a difference of 15.3–12.4 = 2.8 pmol/l (22.6%). This additional 22.6% increase is likely due only to differences in PM10, although the difference between HighPM-nSM and LowPM-nSM was nonsignificant (*p* = 0.09). Among exposed to smoke at baseline, the mean E2 levels was 16.5 pmol/l, resulting in a difference of 16.5–12.4 = 4.1 pmol/l (33.1%), more due to exposure to tobacco smoking (*p* = 0.04). The response level of E2 of the joint exposure was 18.4 pmol/l and it was found to be slightly less than additive. The difference in mean E2 levels between the group of joint exposure and the reference condition was statistically significant at *p* = 0.03. If an additive interaction between PM10 and smoking exists, we would expect to see an outcome level of at least 12.4 + 2.8 + 4.1 = 19.4 pmol/l (when both exposures occur). The separate and combined response of both exposures on mean E2 levels of entire menstrual cycle was plotted on Fig. 3.

### Progesterone profiles across PM10 groups and Total Smoke Exposure groups

The RM-ANCOVA showed that the mean levels of P did not differ according to the exposure to PM10 concentrations (130.4 pmol/l vs. 130.7 pmol/l, between-subjects test, F 1,111 = 0.0003, *p* = 0.99), after controlling for age, active and passive smoking and cycle length. The same RM ANCOVA model did not show significant PM10 group × time interaction term (F 6,666 = 1.39, *p* = 0.21, Table 4).

The same analysis also showed that the mean P during the luteal phase did not vary across Total Smoke Exposure groups (132.2 vs. 129.0 pmol/l; between-subjects test, F 1,111 = 0.04, *p* = 0.84), after controlling for age, PM10 and cycle length. There were also no interactions between Total Smoke Exposure groups and time (between-subjects test, F 6,666 = 1.16, *p* = 0.32), after controlling for age, PM10, and cycle length.

There was no effect modification by Total Smoke Exposure of the association between PM10 groups and mean P (between-subject test, F 1,110 = 0.04, *p* = 0.85) or PM10 groups and P profiles (within-subject test F6,660 = 1.57, *p* = 0.16).

### Estradiol/progesterone ratio profiles between PM10 groups and between Total Smoke Exposure groups

**Fig. 4:**
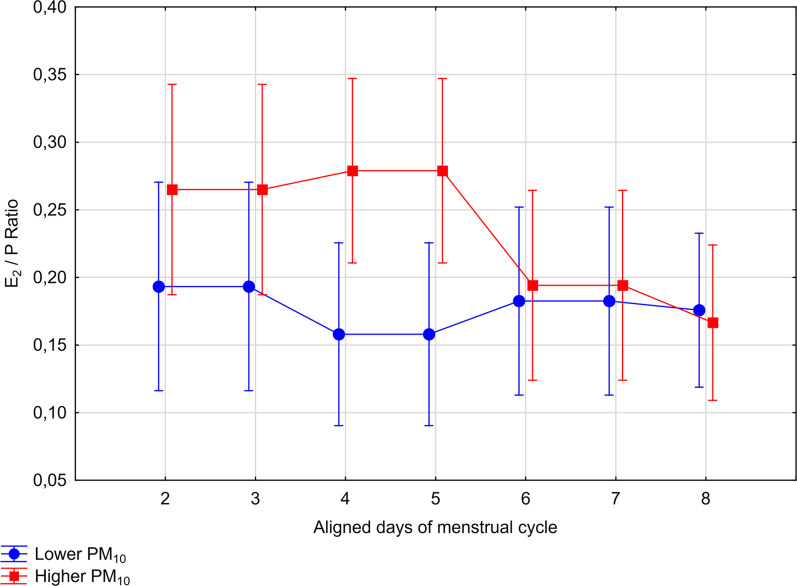
Profiles of E2/P ratios across two groups of regularly menstruating women exposed to low (n = 53) and high (n = 62) levels of PM10, after controlling for age, active and passive smoking and cycle length

After controlling for age, active and passive smoking and cycle length, RM-ANCOVA model showed that the difference between low and high PM10 exposure group in E2/P ratios varied throughout the days of the luteal phase with menstrual cycle days, as pointed in the statistically significant PM10 group × time interaction term (within-subject test, F 6,666 = 4.79, *p* = 0.003, with H–F correction, Table 4). Profiles of E2/P ratios in two groups of women exposed to low and high levels of PM10 are shown in Fig. 4. Next, an exploratory post-hoc analysis were run separately for the early-luteal and the mid-luteal phase. The differences in E2/P ratios between women of high and low exposure to PM10 were seen only in the early-luteal phase of the cycle: the E2/P ratios measured during the days 2–4 of the luteal phase was elevated in a group of women exposed to high PM10 levels in comparison to the group of low exposure to PM10 (F 1,111 = 6.23, *p* = 0.01). There were no differences in E2/P ratios in the mid-luteal phase of the cycle (F 1,111 = 0.70, *p* = 0.40).

The same RM-ANCOVA model showed that the mean E2/P during the luteal phase did not vary across Total Smoke Exposure groups (between-subjects test, F 1,111 = 1.26, *p* = 0.26), after controlling for age, PM10 and cycle length. There was also no interaction between Total Smoke Exposure groups and time (within-subjects test, F 6,666 = 1.43, *p* = 0.23) in explaining E2/P ratios profiles, after controlling to the same confounders (Table 4).

There was no effect modification by Total Smoke Exposure of the association between PM10 groups and mean E2/P (between-subject test, F 1,110 = 0.07, *p* = 0.80) or PM10 groups and E2/P profiles (within-subject test F 6,660 = 1.46, *p* = 0.23), after standardization to age and cycle length (Table 4).

Retrospective repeated measures power analysis based on observed effect sizes found that the existing sample size had sufficient power to determine statistically significant differences among PM or TSE groups in estradiol (average 92% power) but not progesterone profiles (average 51% power), if they existed. As regards interaction effect, actual sample size, had 89% power to determine statistically significant differences among 4 groups of women (PM10 × Total Smoking Exposure) in estradiol profiles. However, the observed power of between-subject effects (determining statistically significant differences among PM or TSE groups in mean hormone levels) was as follows: 52% in estradiol, and only 5% in progesterone levels. This is less than the theoretically desired the conventional value of 80% power [32].

## Discussion

Populations living in urban areas are affected by air pollution, which has become one of the most important humans’ health risk factors [33–35]. In particular many studies have shown that air pollution adversely affects women’s reproductive health including decreased fertility, adverse negative reproductive outcome, low birth weight or enhances the hormone-related cancer [36–39]. The mechanisms are not entirely understood. We suggest that air pollution may cause those health problems through disturbances in the metabolism of E2. To our knowledge, our study is the first that have shown the variation in E2 concentrations across the menstrual cycles of premenopausal women in relation to exposure to different levels of particulate matter.

Although we did not investigate the mechanisms by which PM10 may affect the metabolism of estrogen, it is likely that this is related to the composition of PM10 and its biological activities. More than 90% of compounds that constitute particulate matter belongs to a broad category of hormonally active compounds, namely polycyclic aromatic hydrocarbons (PAHs), and are regarded as EDC [18, 20]. Many studies have shown high correlations between concentrations of PM10 and PM10-bound PAHs [19, 21, 22]. The association between those components in metabolism of estrogen-regulated pathways has been explored [18, 40, 41]. It is thus justified to suggest that the PM in ambient air pollution, which is commonly monitored, can be used as a proxy for PAHs exposure [22, 42, 43].

The mechanisms through which PM10-PAHs induce sex hormone changes are unclear. However, PAHs and their metabolites, which are relatively strong the aryl hydrocarbon receptor (AhR) ligands, exert both anti-estrogenic and estrogenic effects subsequently inhibiting or enhancing estrogenic signaling via crosstalk between AhR and estrogen receptors (ERs) or interfere with the AhR-controlled enzymes [41, 44]. This contradictory effect on metabolism of estrogens strictly depends on PAHs chemical structure and on the diversity of the complex PAHs mixture present in air pollution and in tobacco smoke and their metabolites occur after entering to the body [23, 45–47].

The biosynthesis of estrogens from cholesterol and their elimination involves many enzymes which belong to cytochrome P450 (CYPs) family [48, 49]. Also, PAHs require metabolic activation by cytochrome P450 enzymes. Therefore, higher bioavailability of E2 may be expected when PAHs compete with E2 for the same detoxifying enzymes. However, some other compounds of PAHs family exert the upregulation of those estrogen-catabolizing enzymes which can result in a decrease in E2 hormone levels. It is possible that the effect of PAHs exposure on metabolism of estrogen depends also on the co-stimulation with E2 [50–52].

Another important pathway to alter the rate of estrogen production is by the disturbances in the expression of cytochrome P450 aromatase. This rate-limiting enzyme in estrogen biosynthesis is encoded by the *CYP19* gene in human [53]. It was demonstrated that human breast cells exposed to low-dose environmental endocrine-disrupting compounds, can up-regulate aromatase activity and importantly, increase the intracellular biosynthesis of 17β-estradiol [54, 55]. Comprehensive reviews and discussions of the mechanistic pathways through which that particulate exposure can contribute to the metabolism of sex steroid hormones and female reproductive health are provided elsewhere [24, 56, 57].

Regardless of these mechanisms, a brief exposure to elevated ambient particulate matter may have harmful impact on women’s reproduction. Exposure to environmental pollution during sensitive, critical window was found to induce early pregnancy loss [58, 59] and disturbances in fetal growth, especially during the first trimester [60]. This supports the observation that the uterus is extremely sensitive to estrogen levels during implantation [61]. As such, even small changes in E2 levels related to the exposure to PM10 reported in our study may affect fertility. It has to be noted that the levels of PM10 in Krakow during the period of investigation (June 2001 to June 2003) very often exceeded daily air quality limit value (i. e. 50 µg m^−3^) [62].

Cigarette smoke, beyond the ‘common’ constituents such as nicotine and CO, is a principal source of the components that make up particle-bound PAHs particles [46] The contribution of environmental tobacco smoke (ETC) to outdoor PM in an urban area in southeastern Europe, in particular the quantity of PAHs, was determined by Gini et al. [63]. They underline that the persistence of ETS and possible exposures to significant quantities of tobacco residues outdoors. It was also shown that smoking one cigarette exposes the human respiratory tract to between 10,000 and 40,000 μg PM and that the composition of PM in cigarette smoke is comparable to that of other particles generated through combustion of carbonaceous material found in air pollution [64]. Not only PM in air pollution but also PM in cigarette smoke is a well-established risk factor for many chronic diseases including those related to reproductive hormones levels. Furthermore, epidemiological evidence suggest that cigarette smoke can interfere with steroid hormone transport, storage, metabolism, and clearance, resulting in changes in circulating hormone concentrations [65–67]. Indeed, some studies have found associations between smoking status and estrogen and progesterone [65, 68], while others showed that endogenous estrogen levels do not vary between nonsmokers and smokers [67, 69, 70]. Reasons for those contradictory results are not clear. It is likely that the lack of information in most of the smoke-related research about the brands of cigarettes, size and length of cigarette, manner of smoking and different additives [71, 72], which affect the amount of particle matter and its composition emitted with cigarette smoke and inhaled by smokers and second-hand smokers is responsible for this inconsistency.

Our findings are consistent with studies reporting higher E2 levels among women exposed to tobacco smoke [73, 74]. We found that exposure to cigarettes, either as a passive or active smoker, affects the entire profiles of E2: the mean levels of E2 were significantly higher among the SM group compared with the nonexposed group, after controlling for age, PM10 and cycle length. It was also found that Total Smoke Exposure is not an effect modifier of the association between PM10 and hormone levels or hormone profiles of E2, P or E2/P ratios. This finding suggests that these two harmful environmental contaminants (tobacco smoke and PM10 pollution) affect ovarian hormones separately rather than interactively. It is plausible that other constituents than the compounds in particle matter emanating from cigarette smoke is responsible for the changes in hormonal milieu. Exposure to cigarette smoke affects the entire E2 profile, whilst particulate matter predominantly affects levels of E2 in the early luteal phase of the cycle, when corpus luteum—a temporary endocrine structure in female ovaries—develops from an ovarian follicle. It is possible that tobacco smoke contains different compounds than particulate matter in air pollution which act additionally in evoking estrogenic effect on menstrual cycle physiology. However, our results regarding significant differences in E2 in the early luteal phase were part of a post-hoc, exploratory analysis, and should be interpreted with caution.

Several limitations of the study may be pointed out. First, we found inadequate power to determine statistically significant differences among PM or TSE groups in progesterone levels. This leads to increasing the risk of a type II error. A type II error refers to situation when no statistically significant differences are detected when, in fact, statistically significant differences actually exist. Second limitation is related to grouping of women into high and low categories when studying a continuous exposure. Moreover, grouping of smokers and non-smokers who lived with a smoker may pose a methodological problem.

Thirdly, we used the self-reported measure of exposure to smoke, asking only about the current smoking status (yes or no) and the current exposure to passive smoking (yes or no). It is a quick, straightforward, and inexpensive method of assessment of cigarette use, however it is possible to study exposure to smoking with use of biomarkers such as cotinine (a major metabolite of nicotine) assays [72, 75]. This marker is often viewed as the gold standard for assessing nicotine exposure due to the objectivity of these measures and the decreased susceptibility to reporting bias. However, the high correlation of various self-report measures with this and other biomarkers of smoking showed by others [76, 77] justified our choice of collecting information about the exposure to smoking.

Another limitation is related to methodology of pollution exposure assessment that did not account for variation in a distance from a place of residence and a stationary pollution monitor. However, women living in metropolitan locations are not exposed to pollutants only in their place of living, but also in their workplace, during travel to work, and during all other activities, and thus their exposure might be better represented by the average, city-wide pollution measurement, rather than by just a station closes to home. This problem was also discussed by others [6]. Moreover, the quality of the network of air monitoring stations in Malopolska region and its appropriate coverage of the Krakow residency area is considered to be satisfactory (3–5 monitoring stations), what has been shown by the cohort study conducted on women in the same city [78].

The next methodological problem related to exposure assessment is not taking into consideration indoor air quality, which depends on many factors such as types of windows and the heating system at home. However, the indoor concentration of PM is highly influenced by outdoor air quality [79] and thus allow us to use only outdoor air pollution as an indicator of exposure, especially when air purifiers were not frequently used in Krakow during the time of the study.

A strength of the study was the precise way of assessing the levels of ovarian hormones which was measured in daily saliva samples. In humans, circulating E2 derives predominantly from aromatization in peripheral adipose tissues and 40–60% of the total pool in women circulates bound to sex hormone-binding globulin SHBG circulation [80]. Whereas only 2–3% of estradiol circulates freely and this small percentage of E2 is able to enter the cell and bind to steroid receptors. This free fraction is often considered to be most active and can be measured in saliva by radioimmunoassay. Measurement of sex hormones in saliva provides a noninvasive means of assessing changes throughout the menstrual cycle and allows relatively reliable identification of the menstrual phases as follicular, ovulation and luteal [81]. This method is especially valid in population studies [82], because saliva is one of the most accessible fluids in the human body and it can be obtained frequently, easily, safely, in non-clinical settings, and with low cost. The methodology was validated and results were published previously [81, 83]. The sensitivity and precision of the salivary E_2_ assay make it comparable with assays of serum E_2_ for assessing changes in hormone levels [84]. In our study the radioimmunoassay was used to measure this unbound fraction of β-estradiol and progesterone in each day of the entire menstrual cycle or respectively in luteal phase in salivary samples. At this stage of our study, it is impossible to elucidate the reason why the circulating E2 concentration across the menstrual cycle in women exposed to higher than 54.8 µg/m^3^ PM10 is enhanced as compared to women exposed to lower levels of PM10. However, additionally to the reasons mentioned above, the unbound estrogen concentrations may be elevated in polluted environment due to the competition between PAHs with endogenous estrogen for binding to SHBG. It was shown that some xenoestrogens were able to dose-dependently increase concentrations of hSHBG-unbound testosterone and/or estradiol in native plasma from normal men and women [85].

## Conclusions

This is the first study that has directly examined the associations of PM10 and cigarette smoke with measures of daily changes across menstrual cycle in premenopausal women. We suggest that reproductive hormone concentrations are very sensitive to air pollution. The health consequences of this, especially the enhancement of E2 and in the ratio between E2/P in women exposed to the higher than European daily norm of PM10 discussed above, should not be ignored, given that the air pollution is one of the less modifiable lifestyle variables. Furthermore, most people living in highly polluted areas do not have the opportunity of moving to a less polluted areas. Therefore, it is possible that reproductive age women, exposed to a high level of PM10-PAHs, or smoking (passively or actively), have a higher risk of hormone-related cancers and reproductive health problems due to enhanced E2 levels and imbalance in sex-hormone. The results also underline the importance of pre-conception counseling couples planning pregnancy making them aware that this period of human reproduction is particularly sensitive to the quality of the environment.

## Data Availability

The datasets used and/or analysed during the current study are available from the corresponding author on reasonable request.

## Abbreviations

AhR: Aryl hydrocarbon receptor
CYP 19: Cytochrome P450
DNA: Deoxyribonucleic acid
EDC: Endocrine disrupting chemicals
E2: 17β-Estradiol
H–F adjustment: Huynh and Feldt adjustment, a correction for violations of sphericity
IQR: Interquartile range (Q1–Q3)
nSM: Non-exposed to smoke (not being exposed neither as an active nor passive smoker)
PM10: Particulate matter with particles of aerodynamic diameter less than 10 µm
PAHs: Polycyclic aromatic hydrocarbons
P: Progesterone
TSE: Total Smoke Exposure
RM-ANCOVA: Repeated measures analysis of covariance
SM: Exposed to smoke (being exposed either as an active or passive smoker)

## References

[1] Lelieveld J, Pozzer A, Poschl U, Fnais M, Haines A, Munzel T (2020). Loss of life expectancy from air pollution compared to other risk factors: a worldwide perspective. Cardiovasc Res.

[2] Reitsma MB, Kendrick PJ, Ababneh E, Abbafati C, Abbasi-Kangevari M, Abdoli A, Abedi A, Abhilash ES, Abila DB, Aboyans V, Abu-Rmeileh NM (2021). Spatial, temporal, and demographic patterns in prevalence of smoking tobacco use and attributable disease burden in 204 countries and territories, 1990–2019: a systematic analysis from the Global Burden of Disease Study 2019. Lancet.

[3] Jedrychowski WA, Majewska R, Spengler JD, Camann D, Roen EL, Perera FP (2017). Prenatal exposure to fine particles and polycyclic aromatic hydrocarbons and birth outcomes: a two-pollutant approach. Int Arch Occup Environ Health.

[4] Merklinger-Gruchala A, Jasienska G, Kapiszewska M (2017). Effect of air pollution on menstrual cycle length-a prognostic factor of women's reproductive health. Int J Environ Res Public Health.

[5] Bolden AL, Rochester JR, Schultz K, Kwiatkowski CF (2017). Polycyclic aromatic hydrocarbons and female reproductive health: a scoping review. Reprod Toxicol.

[6] Gouveia N, Bremner SA, Novaes HM (2004). Association between ambient air pollution and birth weight in Sao Paulo, Brazil. J Epidemiol Community Health.

[7] Dechanet C, Brunet C, Anahory T, Hamamah S, Hedon B, Dechaud H (2011). Effects of cigarette smoking on female reproduction: from oocyte to embryo (part I). Gynecol Obstet Fertil.

[8] Prior JC (2020). Women’s reproductive system as balanced estradiol and progesterone actions—a revolutionary, paradigm-shifting concept in women’s health. Drug Discov Today Disease Models.

[9] Shalom-Paz E, Aslih N, Samara N, Michaeli M, Ellenbogen A (2015). Late follicular progesterone to estradiol ratio is not influenced by protocols or gonadotropins used. Reprod Biol Endocrinol.

[10] Mascarenhas M, Kamath MS, Chandy A, Kunjummen AT (2015). Progesterone/estradiol ratio as a predictor in the ART cycles with premature progesterone elevation on the day of hCG trigger. J Reprod Infertil.

[11] Band PR, Le ND, Fang R, Deschamps M (2002). Carcinogenic and endocrine disrupting effects of cigarette smoke and risk of breast cancer. Lancet.

[12] Hecht SS (2002). Tobacco smoke carcinogens and breast cancer. Environ Mol Mutagen.

[13] Jasienska G, Thune I (2001). Lifestyle, hormones, and risk of breast cancer. BMJ.

[14] Rosenberg CR, Pasternack BS, Shore RE, Koenig KL, Toniolo PG (1994). Premenopausal estradiol levels and the risk of breast cancer: a new method of controlling for day of the menstrual cycle. Am J Epidemiol.

[15] Tansavatdi K, McClain B, Herrington DM (2004). The effects of smoking on estradiol metabolism. Minerva Ginecol.

[16] Windham GC, Mitchell P, Anderson M, Lasley BL (2005). Cigarette smoking and effects on hormone function in premenopausal women. Environ Health Perspect.

[17] Teil MJ, Moreau-Guigon E, Blanchard M, Alliot F, Gasperi J, Cladiere M, Mandin C, Moukhtar S, Chevreuil M (2016). Endocrine disrupting compounds in gaseous and particulate outdoor air phases according to environmental factors. Chemosphere.

[18] Wenger D, Gerecke AC, Heeb NV, Schmid P, Hueglin C, Naegeli H, Zenobi R (2009). In vitro estrogenicity of ambient particulate matter: contribution of hydroxylated polycyclic aromatic hydrocarbons. J Appl Toxicol.

[19] Ravindra K, Mittal AK, Van Grieken R (2001). Health risk assessment of urban suspended particulate matter with special reference to polycyclic aromatic hydrocarbons: a review. Rev Environ Health.

[20] Bonner MR, Han D, Nie J, Rogerson P, Vena JE, Muti P, Trevisan M, Edge SB, Freudenheim JL (2005). Breast cancer risk and exposure in early life to polycyclic aromatic hydrocarbons using total suspended particulates as a proxy measure. Cancer Epidemiol Biomark Prev.

[21] Bostrom CE, Gerde P, Hanberg A, Jernstrom B, Johansson C, Kyrklund T, Rannug A, Tornqvist M, Victorin K, Westerholm R (2002). Cancer risk assessment, indicators, and guidelines for polycyclic aromatic hydrocarbons in the ambient air. Environ Health Perspect.

[22] Akyuz M, Cabuk H (2009). Meteorological variations of PM2.5/PM10 concentrations and particle-associated polycyclic aromatic hydrocarbons in the atmospheric environment of Zonguldak, Turkey. J Hazard Mater.

[23] Bekki K, Toriba A, Tang N, Kameda T, Hayakawa K (2013). Biological effects of polycyclic aromatic hydrocarbon derivatives. J Uoeh.

[24] Santodonato J (1997). Review of the estrogenic and antiestrogenic activity of polycyclic aromatic hydrocarbons: relationship to carcinogenicity. Chemosphere.

[25] White AJ, Bradshaw PT, Hamra GB (2018). Air pollution and breast cancer: a review. Curr Epidemiol Rep.

[26] Akhmedkhanov A, Zeleniuch-Jacquotte A, Toniolo P (2001). Role of exogenous and endogenous hormones in endometrial cancer: review of the evidence and research perspectives. Ann N Y Acad Sci.

[27] Schindler AE (2009). Progestogen deficiency and endometrial cancer risk. Maturitas.

[28] Lipson SF, Ellison PT (1996). Comparison of salivary steroid profiles in naturally occurring conception and non-conception cycles. Hum Reprod.

[29] Jasienska G, Ziomkiewicz A, Thune I, Lipson SF, Ellison PT (2006). Habitual physical activity and estradiol levels in women of reproductive age. Eur J Cancer Prev.

[30] Jasienska G, Ellison PT (2004). Energetic factors and seasonal changes in ovarian function in women from rural Poland. Am J Hum Biol.

[31] Vandenbroucke JP, von Elm E, Altman DG, Gotzsche PC, Mulrow CD, Pocock SJ, Poole C, Schlesselman JJ, Egger M (2007). Strengthening the Reporting of Observational Studies in Epidemiology (STROBE): explanation and elaboration. Epidemiology.

[32] Cohen J (1992). A power primer. Psychol Bull.

[33] Aleksandropoulou V, Eleftheriadis K, Diapouli E, Torseth K, Lazaridis M (2012). Assessing PM10 source reduction in urban agglomerations for air quality compliance. J Environ Monit.

[34] Amarillo AC, Tavera Busso I, Carreras H (2014). Exposure to polycyclic aromatic hydrocarbons in urban environments: health risk assessment by age groups. Environ Pollut.

[35] Bostrom CE, Almen J, Steen B, Westerholm R (1994). Human exposure to urban air pollution. Environ Health Perspect.

[36] Wu J, Laurent O, Li L, Hu J, Kleeman M (2016). Adverse reproductive health outcomes and exposure to gaseous and particulate-matter air pollution in pregnant women. Res Rep Health Eff Inst.

[37] Conforti A, Mascia M, Cioffi G, De Angelis C, Coppola G, De Rosa P, Pivonello R, Alviggi C, De Placido G (2018). Air pollution and female fertility: a systematic review of literature. Reprod Biol Endocrinol.

[38] Sram R (1999). Impact of air pollution on reproductive health. Environ Health Perspect.

[39] Zhang Y, Yang J, Ma L (2017). The influence of ambient air pollution on reproductive health: current situation and prospects. Zhonghua Yu Fang Yi Xue Za Zhi.

[40] Shimada T, Murajama N, Tanaka K, Takenaka S, Imai Y, Hopkins NE, Foroozesh MK, Alworth WL, Yamazaki H, Guengerich FP (2008). Interaction of polycyclic aromatic hydrocarbons with human cytochrome P450 1B1 in inhibiting catalytic activity. Chem Res Toxicol.

[41] Tarnow P, Tralau T, Luch A (2019). Chemical activation of estrogen and aryl hydrocarbon receptor signalling pathways and their interaction in toxicology and metabolism. Expert Opin Drug Metab Toxicol.

[42] Najmeddin A, Keshavarzi B (2019). Health risk assessment and source apportionment of polycyclic aromatic hydrocarbons associated with PM10 and road deposited dust in Ahvaz metropolis of Iran. Environ Geochem Health.

[43] Wiriya W, Prapamontol T, Chantara S (2013). PM10-bound polycyclic aromatic hydrocarbons in Chiang Mai (Thailand): seasonal variations, source identification, health risk assessment and their relationship to air-mass movement. Atmos Res.

[44] Matthews J, Gustafsson JA (2006). Estrogen receptor and aryl hydrocarbon receptor signaling pathways. Nucl Recept Signal.

[45] Gozgit JM, Nestor KM, Fasco MJ, Pentecost BT, Arcaro KF (2004). Differential action of polycyclic aromatic hydrocarbons on endogenous estrogen-responsive genes and on a transfected estrogen-responsive reporter in MCF-7 cells. Toxicol Appl Pharmacol.

[46] Kamiya M, Toriba A, Onoda Y, Kizu R, Hayakawa K (2005). Evaluation of estrogenic activities of hydroxylated polycyclic aromatic hydrocarbons in cigarette smoke condensate. Food Chem Toxicol.

[47] Shimada T, Guengerich FP (2006). Inhibition of human cytochrome P450 1A1-, 1A2-, and 1B1-mediated activation of procarcinogens to genotoxic metabolites by polycyclic aromatic hydrocarbons. Chem Res Toxicol.

[48] Shoham Z, Schachter M (1996). Estrogen biosynthesis—regulation, action, remote effects, and value of monitoring in ovarian stimulation cycles. Fertil Steril.

[49] Payne AH, Hales DB (2004). Overview of steroidogenic enzymes in the pathway from cholesterol to active steroid hormones. Endocr Rev.

[50] Christou M, Savas U, Schroeder S, Shen X, Thompson T, Gould MN, Jefcoate CR (1995). Cytochromes CYP1A1 and CYP1B1 in the rat mammary gland: cell-specific expression and regulation by polycyclic aromatic hydrocarbons and hormones. Mol Cell Endocrinol.

[51] Son DS, Roby KF, Rozman KK, Terranova PF (2002). Estradiol enhances and estriol inhibits the expression of CYP1A1 induced by 2,3,7,8-tetrachlorodibenzo-p-dioxin in a mouse ovarian cancer cell line. Toxicology.

[52] Ricci MS, Toscano DG, Mattingly CJ, Toscano WA (1999). Estrogen receptor reduces CYP1A1 induction in cultured human endometrial cells. J Biol Chem.

[53] Thomas MP, Potter BV (2013). The structural biology of oestrogen metabolism. J Steroid Biochem Mol Biol.

[54] Williams GP, Darbre PD (2019). Low-dose environmental endocrine disruptors, increase aromatase activity, estradiol biosynthesis and cell proliferation in human breast cells. Mol Cell Endocrinol.

[55] Saito R, Miki Y, Hata S, Ishida T, Suzuki T, Ohuchi N, Sasano H (2017). Aryl hydrocarbon receptor induced intratumoral aromatase in breast cancer. Breast Cancer Res Treat.

[56] Johansson HKL, Damdimopoulou P, van Duursen MBM, Boberg J, Franssen D, de Cock M, Jaager K, Wagner M, Velthut-Meikas A, Xie Y (2020). Putative adverse outcome pathways for female reproductive disorders to improve testing and regulation of chemicals. Arch Toxicol.

[57] Hernandez-Ochoa I, Karman BN, Flaws JA (2009). The role of the aryl hydrocarbon receptor in the female reproductive system. Biochem Pharmacol.

[58] Perin PM, Maluf M, Czeresnia CE, Januario DA, Saldiva PH (2010). Impact of short-term preconceptional exposure to particulate air pollution on treatment outcome in couples undergoing in vitro fertilization and embryo transfer (IVF/ET). J Assist Reprod Genet.

[59] Enkhmaa D, Warburton N, Javzandulam B, Uyanga J, Khishigsuren Y, Lodoysamba S, Enkhtur S, Warburton D (2014). Seasonal ambient air pollution correlates strongly with spontaneous abortion in Mongolia. BMC Pregnancy Childbirth.

[60] Merklinger-Gruchala A, Kapiszewska M (2015). Association between PM10 air pollution and birth weight after full-term pregnancy in Krakow city 1995–2009—trimester specificity. Ann Agric Environ Med.

[61] Ma WG, Song H, Das SK, Paria BC, Dey SK (2003). Estrogen is a critical determinant that specifies the duration of the window of uterine receptivity for implantation. Proc Natl Acad Sci U S A.

[62] Reizer M, Juda-Rezler K (2016). Explaining the high PM10 concentrations observed in Polish urban areas. Air Qual Atmos Health.

[63] Gini M, Lianou M, Chalbot MC, Kotronarou A, Kavouras IG, Helmis CG (2013). Quantification of environmental tobacco smoke contribution on outdoor particulate aliphatic and polycyclic aromatic hydrocarbons. Arch Environ Contam Toxicol.

[64] National Research Council (1986). Environmental tobacco smoke: measuring exposures and assessing health effects.

[65] Soldin OP, Makambi KH, Soldin SJ, O'Mara DM (2011). Steroid hormone levels associated with passive and active smoking. Steroids.

[66] Thomas EJ, Edridge W, Weddell A, McGill A, McGarrigle HH (1993). The impact of cigarette smoking on the plasma concentrations of gonadotrophins, ovarian steroids and androgens and upon the metabolism of oestrogens in the human female. Hum Reprod.

[67] Whitcomb BW, Bodach SD, Mumford SL, Perkins NJ, Trevisan M, Wactawski-Wende J, Liu A, Schisterman EF (2010). Ovarian function and cigarette smoking. Paediatr Perinat Epidemiol.

[68] Sterzik K, Strehler E, De Santo M, Trumpp N, Abt M, Rosenbusch B, Schneider A (1996). Influence of smoking on fertility in women attending an in vitro fertilization program. Fertil Steril.

[69] Berta L, Frairia R, Fortunati N, Fazzari A, Gaidano G (1992). Smoking effects on the hormonal balance of fertile women. Horm Res.

[70] Key TJ, Pike MC, Baron JA, Moore JW, Wang DY, Thomas BS, Bulbrook RD (1991). Cigarette smoking and steroid hormones in women. J Steroid Biochem Mol Biol.

[71] Kant N, Müller R, Braun M, Gerber A, Groneberg D (2016). Particulate matter in second-hand smoke emitted from different cigarette sizes and types of the brand vogue mainly smoked by women. Int J Environ Res Public Health.

[72] Rebagliato M (2002). Validation of self reported smoking. J Epidemiol Community Health.

[73] Zumoff B, Miller L, Levit CD, Miller EH, Heinz U, Kalin M, Denman H, Jandorek R, Rosenfeld RS (1990). The effect of smoking on serum progesterone, estradiol, and luteinizing hormone levels over a menstrual cycle in normal women. Steroids.

[74] Brand JS, Chan MF, Dowsett M, Folkerd E, Wareham NJ, Luben RN, van der Schouw YT, Khaw KT (2011). Cigarette smoking and endogenous sex hormones in postmenopausal women. J Clin Endocrinol Metab.

[75] Benowitz NL (1996). Cotinine as a biomarker of environmental tobacco smoke exposure. Epidemiol Rev.

[76] Wray JM, Gass JC, Miller EI, Wilkins DG, Rollins DE, Tiffany ST (2016). A comparative evaluation of self-report and biological measures of cigarette use in nondaily smokers. Psychol Assess.

[77] Vartiainen E, Seppala T, Lillsunde P, Puska P (2002). Validation of self reported smoking by serum cotinine measurement in a community-based study. J Epidemiol Community Health.

[78] Jedrychowski W, Bendkowska I, Flak E, Penar A, Jacek R, Kaim I, Spengler JD, Camann D, Perera FP (2004). Estimated risk for altered fetal growth resulting from exposure to fine particles during pregnancy: an epidemiologic prospective cohort study in Poland. Environ Health Perspect.

[79] Scibor M, Galbarczyk A, Jasienska G (2019). Living well with pollution? The impact of the concentration of PM2.5 on the quality of life of patients with asthma. Int J Environ Res Public Health.

[80] Rosner W (2015). Free estradiol and sex hormone-binding globulin. Steroids.

[81] Gandara BK, Leresche L, Mancl L (2007). Patterns of salivary estradiol and progesterone across the menstrual cycle. Ann N Y Acad Sci.

[82] Jasienska G, Jasienski M (2008). Interpopulation, interindividual, intercycle, and intracycle natural variation in progesterone levels: a quantitative assessment and implications for population studies. Am J Hum Biol.

[83] Chatterton RT, Mateo ET, Hou N, Rademaker AW, Acharya S, Jordan VC, Morrow M (2005). Characteristics of salivary profiles of oestradiol and progesterone in premenopausal women. J Endocrinol.

[84] Celec P, Ostanikova D, Skoknova M, Hodosy J, Putz Z, Kudela M (2009). Salivary sex hormones during the menstrual cycle. Endocr J.

[85] Dechaud H, Ravard C, Claustrat F, de la Perriere AB, Pugeat M (1999). Xenoestrogen interaction with human sex hormone-binding globulin (hSHBG). Steroids.

